# Prediction of lymphovascular invasion in non-mass enhancement breast cancer using DCE-MRI and clinical-pathological features

**DOI:** 10.3389/fonc.2025.1539748

**Published:** 2025-10-06

**Authors:** Shiqi Guo, Kai Zhao, Hu Xu, Yujiao Xie, Qingyang Li, Junqing Liang, Siyi Chen, Jiahong Sun, Zhaofeng Gao, Li Zhu, Jiandong Wang

**Affiliations:** ^1^ Department of General Surgery, The First Medical Center of Chinese People's Liberation Army General Hospital, Beijing, China; ^2^ School of Medicine Nankai University, Tianjin, China; ^3^ Department of Breast Surgery, Affiliated Cancer Hospital of Inner Mongolia Medical University, Hohhot, Inner Mongolia, China

**Keywords:** lymphovascular invasion, molecular subtype, non-mass enhancement, DCE-MRI, invasive breast cancer

## Abstract

**Objective:**

The present study explores the relationship between the distribution patterns of non-mass enhancement (NME) type invasive breast cancer in dynamic contrast-enhanced magnetic resonance imaging (DCE-MRI) and lymphovascular invasion (LVI).

**Methods:**

A retrospective analysis was conducted on 192 female patients with NME-type breast cancer who underwent DCE-MRI between January 2019 and December 2023. Based on postoperative pathological results, the patients were divided into two groups: LVI-positive [LVI(+)] (N = 50) and LVI-negative [LVI(-)] (N = 142). A generalized variance inflation factor (GVIF) analysis was used to identify variables with high multicollinearity. Multivariate logistic regression was used to analyze the risk factors associated with LVI. The performance of the Distribution of NME + ADC + Molecular subtype was evaluated using receiver operating characteristic (ROC) curves and the areas under the curve (AUC). A nomogram was built based on the predictive factors and internally evaluated using a bootstrap resampling method (1000 bootstrap resamples). The performance of the predictive model was evaluated by calibration curve and decision curve analysis (DCA). The DeLong test was applied to compare differences between AUC values, while net reclassification improvement (NRI) and integrated discrimination improvement (IDI) were used to assess the predictive ability of adding the Distribution of NME to the basic model [apparent diffusion coefficient (ADC) + Molecular subtype)].

**Results:**

Compared to focal distribution, patients with linear distribution of NME had a higher risk of LVI positivity (*P* = 0.030). Distribution of NME + ADC + Molecular subtype demonstrated a relatively strong ability to predict LVI status, with an AUC of 0.723. Compared to the performance of each risk factor alone in predicting LVI, the differences in AUC were statistically significant (*P* = 0.008, *P* = 0.006, *P* = 0.012, DeLong test). Additionally, the inclusion of Distribution of NME could effectively improve the ability of basic model (ADC + Molecular subtype) to predict LVI, its NRI value was 0.389 (*P* = 0.013) and its IDI value was 0.047 (*P* = 0.008).

**Conclusion:**

Distribution of NME + ADC + Molecular subtype was effective in predicting LVI status, with an AUC of 0.723. The inclusion of Distribution of NME significantly improved its predictive ability for LVI.

## 1 Introduction

According to GLOBOCAN 2022, breast cancer remains the most common cancer among women worldwide, and imaging examinations play a crucial role in the early detection, diagnosis, and treatment of breast cancer. Dynamic Contrast-Enhanced Magnetic Resonance Imaging (DCE-MRI) has the highest diagnostic accuracy among breast imaging techniques and can be used for preoperative evaluation, treatment planning, and assessing chemotherapy response in breast lesions (1). According to the fifth edition of the Breast Imaging Reporting and Data System (BI-RADS) lexicon, lesions on DCE-MRI are classified into mass enhancement (ME) and non-mass enhancement (NME) (2). NME is a unique imaging feature, characterized by enhancement within fibro glandular tissue without a mass effect, and occurs less frequently than ME.

Lymphovascular invasion (LVI) is a precursor of regional lymph node metastasis in invasive breast cancer and an independent prognostic factor for distant metastasis (3–5). It is significantly associated with poor prognosis and thus plays a crucial role in influencing treatment decisions for breast cancer. LVI is typically detected in postoperative pathological specimens, but preoperative treatments can affect the evaluation of it (6).

Currently, the vast majority of research on NME lesions focuses on predicting malignancy or exploring ways to reduce unnecessary biopsies, there are still few studies examining the factors influencing LVI in NME-type breast cancer (7). Conventional LVI prediction models (e.g., radiomic or clinicopathological frameworks) primarily rely on morphological features of mass-type lesions—such as spiculated margins and enhancement heterogeneity. These characteristics manifest as regional distribution patterns rather than focal masses in NME-type lesions, resulting in significantly diminished predictive performance of traditional models (8, 9). Standard MRI radiomic approaches depend on texture features (e.g., gray-level co-occurrence matrices), yet the diffuse distribution of NME lesions compromises texture feature extraction due to low signal-to-noise ratios and substantial interference from background parenchymal enhancement (BPE) (10).

Therefore, our study will investigate the associations between DCE-MRI imaging features, clinicopathological features, and LVI in NME-type invasive breast cancer patients. We will establish a dedicated NME-specific LVI prediction framework, aiming to provide clinical decision support tools for personalized surgical navigation and evidence-based treatment optimization.

## 2 Materials and methods

### 2.1 Patients

This study was a retrospective case-control study, involving 192 patients with non-mass enhancement (NME) invasive breast cancer who underwent DCE-MRI between January 2019 and December 2023. Based on postoperative pathological results, the patients were divided into two groups: LVI-positive [LVI(+)] (N = 50) and LVI-negative [LVI(-)] (N = 142). Inclusion criteria included: a) Female patients aged ≥18 years with pathologically confirmed invasive breast cancer after surgery; b) Patients who underwent DCE-MRI at our center prior to surgery, and whose imaging showed non-mass enhancement; c) Patients who had not received systemic or local treatments, including neoadjuvant therapy, radiotherapy, biopsy or surgery, within two years before the DCE-MRI examination. Exclusion criteria included: a) Patients with inflammatory breast cancer; b) Patients lacking key imaging parameters, clinical-pathological information, or follow-up data; c) Patients with concurrent other malignancies; d) Patients with pregnancy or a history of metformin use. This study was approved by the Ethics Review Committee of the hospital.

### 2.2 MRI examinations

A 3.0-Tesla MRI scanner with an 8-channel phased array breast coil was used to conduct the MRI examinations (Discovery 750, GE HealthCare, Chicago, IL, USA). The imaging protocol, lasting for 18 minutes, included 4 pulse sequences: diffusion-weighted imaging (DWI), T2-weighted imaging (T2WI), T1-weighted imaging (T1WI), and DCE. All sequences were spatially matched in axial view, with a field of view of 320 mm × 320 mm and 190 mm of z-axis coverage. The b value of DWI was 0 and 1,000 s/mm^2^ in 3 orthogonal diffusion gradients, the inversion recovery (IR) was 250 ms for fat suppression, the repetition time (TR) was 5,400 ms, minimum echo time (TE), and the matrix size was 128×128. The T2WI used iterative decomposition of water and fat with echo asymmetric and least squares estimation (IDEAL) for fat suppression, with a TR of 5,000 ms, a TE of 68 ms, and a matrix size of 320×256. The T1WI and DCE had the same geometric location and were performed with the following parameters: an isotropic spatial resolution of 1.0 mm × 1.0 mm × 1.0 mm, 192 partitions in the axial view, a minimum TR/TE, and a flip angle of 120°. The DCE scan repeated 6 continuous phases without interruption, each of which lasted 120 seconds (s). Following the pre-contrast phase, 0.5 M of gadopentetic acid (Gd-DTPA) was administered into the antecubital vein at a rate of 2 mL/s and a dose of 0.1 mmol/kg body weight and was followed by a 20-mL flush of saline. The time-intensity curve (TIC) was classified using the enhancement ratio difference between the first-enhanced phase and the last-enhanced phase (8–10 minutes after contrast delivery). The persistent, plateau and washout TICs were defined as enhancement ratios of <−10%, −10% to 10%, and >10%, respectively.

### 2.3 DCE-MRI imaging features

The study used a blinded diagnostic approach. Each patient’s imaging data were independently reviewed and evaluated by two radiologists specializing in breast diseases, with 8 and 12 years of experience, respectively, and formal professional training. Both radiologists independently interpreted and assessed the DCE-MRI images without knowledge of the patient’s clinical data, other test results, or treatment measures.

The description of imaging features followed the fifth edition of the BI-RADS lexicon (2).NME refers to enhancement detected in fibroglandular tissue regions on DCE-MRI without a mass effect. NME is categorized based on internal enhancement patterns (homogeneous, heterogeneous, clustered ring, and clumped enhancement) and distribution patterns (focal, linear, segmental, regional, multiple regions, and diffuse enhancement). Background parenchymal enhancement (BPE) refers to the enhancement of normal breast parenchyma and is classified as mild, moderate, or marked. The apparent diffusion coefficient (ADC) is a parameter describing the rate and extent of water molecule diffusion in DWI. The rate of early enhancement is calculated using the formula: (post-enhancement signal intensity - pre-enhancement signal intensity) × 100%/pre-enhancement signal intensity.

### 2.4 Histopathological evaluation

All histopathological assessments were performed on surgical specimens. Rosen’s histopathological criteria defined LVI as the presence of tumor emboli in the lymphatic and vascular systems. Immunohistochemistry (IHC) Staining: Estrogen receptor (ER) high expression is defined as ≥10% of tumor cell nuclei showing positive staining, while low or no expression is defined as <10% of nuclei staining positive. Progesterone receptor (PR) high expression is similarly defined as ≥10% positive staining in tumor cell nuclei, with low or no expression defined as <10%. Ki-67 is considered highly expressed if >14%, and low expression if ≤14%.

Human epidermal growth factor receptor 2 (HER2) expression is classified based on the proportion and intensity of membrane staining: “Negative” (“-”) refers to no staining or ≤10% of invasive cancer cells showing incomplete and faint membrane staining; “1+” refers to >10% of invasive cancer cells with incomplete and weak membrane staining; “2+” indicates >10% of invasive cancer cells with weak to moderate complete membrane staining or ≤10% of cells with strong complete membrane staining. For patients with “2+” HER2 expression, fluorescence *in situ* hybridization (FISH) is performed to determine HER2 gene amplification; “3+” indicates >10% of invasive cancer cells with strong, complete, and uniform membrane staining. HER2 status is defined as follows (11):

- HER2-0: IHC score of “0”
- HER2-low: IHC score of “1+” or “2+” with a negative FISH result
- HER2-positive: IHC score of “3+” or “2+” with a positive FISH result

According to the 2017 St. Gallen International Breast Cancer Conference consensus (12), breast cancer is classified into four subtypes. Luminal A: ER/PR positive, HER2 negative, and Ki-67 ≤14%; Luminal B: Includes HER2 negative type (ER/PR positive, HER2 negative, and Ki-67 >14%) and HER2 positive type (ER/PR positive, HER2 positive, and any Ki-67 level); HER2 overexpression: ER/PR negative, HER2 positive, and any Ki-67 level; Triple-negative: ER, PR, and HER2 all negative, with any Ki-67 level.

### 2.5 Statistical analysis

Data analysis was performed using R version 4.4.0. All statistical tests were two-sided. Continuous variables were expressed as mean ± standard deviation (SD) or as median and interquartile range (IQR), depending on data distribution. Comparisons of continuous variables between the two groups were conducted using the Student’s t-test or the Mann-Whitney U test. Categorical variables were presented as counts and percentages, and comparisons between the two groups were made using the chi-square (χ^2^) test or Fisher’s exact test. A generalized variance inflation factor (GVIF) analysis was used to identify variables with high multicollinearity. Risk factors for LVI formation in patients with invasive breast cancer were analyzed using multivariate logistic regression. The diagnostic efficacy of each risk factor was evaluated by receiver operating characteristic (ROC) curves and the area under the curve (AUC). A nomogram was built based on the predictive factors and internally evaluated using a bootstrap resampling method (1000 bootstrap resamples). The performance of the predictive model was evaluated by calibration curve. Applying decision curve analysis (DCA) to evaluate the clinical usefulness of the model. DeLong test was used to compare differences between AUC values. Net reclassification improvement (NRI) and integrated discrimination improvement (IDI) were used to assess the predictive ability and clinical benefit of adding the Distribution of NME to the basic model (ADC + Molecular subtype). P-value below 0.05 was considered statistically significant.

## 3 Results

### 3.1 Baseline characteristics

A total of 192 breast cancer lesions from 192 patients were included in this study. Of the patients, 73 (38%) were younger than 45 years, and 177 (92.2%) had invasive ductal carcinoma as their pathological type, other non-ductal forms of invasive breast cancer were classified as papillary carcinoma (10 cases), mucinous carcinoma (3 cases), and invasive lobular carcinoma (2 cases). Due to the limited overall number of cases for these subtypes, they were collectively grouped under the category “Others” for statistical analysis. The median size of the invasive cancers in all patients was 2.0 cm (range: 1.5–3.0 cm). Baseline characteristics are shown in **Table 1**.

**Table 1:** Clinical-pathological and DCE-MRI features of patients with breast cancer between LVI(+) and LVI(-) (n, %).

Features	LVI(-) N=142	LVI(+) N=50	All N=192	*P* value
Clinicopathological features				
Age				**0.028**
<45 yrs	47 (33.1)	26 (52.0)	73 (38.0)	
≥45 yrs	95 (66.9)	24 (48.0)	119 (62.0)	
Tumour type				1.000
IDC	131 (92.3)	46 (92.0)	177 (92.2)	
Others	11 (7.7)	4 (8.0)	15 (7.8)	
Tumor site				0.327
Left	73 (51.41%)	21 (42.00%)	94 (48.96%)	
Right	69 (48.59%)	29 (58.00%)	98 (51.04%)	
Invasive carcinoma size [M(Q1~Q3), cm]	2.0 (1.4~3.0)	2.7 (1.5~4.0)	2.0 (1.5~3.0)	**0.007**
pT (TNM)				**0.030**
1	81 (57.1)	21 (42.0)	102 (53.1)	
2	53 (37.3)	20 (40.0)	73 (38.0)	
3	6 (4.2)	8 (16.0)	14 (7.3)	
4	2 (1.4)	1 (2.0)	3 (1.6)	
ER status				0.067
Low	33 (23.2)	19 (38.0)	52 (27.1)	
High	109 (76.8)	31 (62.0)	140 (72.9)	
PR status				0.142
Low	29 (20.4)	16 (32.0)	45 (23.4)	
High	113 (79.6)	34 (68.0)	147 (76.6)	
Ki-67 status				**0.013**
Low	29 (20.4)	2 (4.0)	31 (16.2)	
High	113 (79.6)	48 (96.0)	161 (83.8)	
HER2 status				0.649
HER2-0	14 (9.8)	3 (6.0)	17 (8.8)	
HER2-low	88 (62.0)	30 (60.0)	118 (61.5)	
HER2-positive	40 (28.2)	17 (34.0)	57 (29.7)	
Molecular subtype				**0.019**
Luminal A	28 (19.7)	2 (4.0)	30 (15.6)	
Luminal B	88 (62.0)	33 (66.0)	121 (63.0)	
HER2-overexpression	18 (12.7)	9 (18.0)	27 (14.1)	
Triple-negative	8 (5.6)	6 (12.0)	14 (7.3)	
Nerve invasion				1.000
Negative	136 (95.8)	48 (96.0)	184 (95.8)	
Positive	6 (4.2)	2 (4.0)	8 (4.2)	
SBR grade				0.696
1	13 (9.2)	4 (8.0)	17 (8.8)	
2	113 (79.6)	38 (76.0)	151 (78.7)	
3	16 (11.2)	8 (16.0)	24 (12.5)	
ALN status				**<0.001**
Negative	87 (61.3)	10 (20.0)	97 (50.5)	
Positive	55 (38.7)	40 (80.0)	95 (49.5)	
pN (TNM)				**<0.001**
0	87 (61.3)	10 (20.0)	97 (50.5)	
1	32 (22.5)	14 (28.0)	46 (24.0)	
2	12 (8.4)	9 (18.0)	21 (10.9)	
3	11 (7.8)	17 (34.0)	28 (14.6)	
DCE-MRI features				
Internal enhancement				
Homogeneous	2 (1.4)	0 (0.0)	2 (1.0)	1.000
Heterogeneous	48 (33.8)	23 (46.0)	71 (37.0)	0.172
Consecutive nodular	32 (22.5)	12 (24.0)	44 (22.9)	0.987
Agglomerated annularly	89 (62.7)	28 (56.0)	117 (60.9)	0.507
Distribution				0.070
Focal	16 (11.3)	2 (4.0)	18 (9.4)	
Linear	4 (2.8)	3 (6.0)	7 (3.7)	
Segmental	83 (58.5)	24 (48.0)	107 (55.7)	
Regional	18 (12.7)	8 (16.0)	26 (13.5)	
Multiple regional	11 (7.8)	3 (6.0)	14 (7.3)	
Diffuse	10 (7.0)	10 (20.0)	20 (10.4)	
BPE				0.697
Minimal	28 (19.7)	11 (22.0)	39 (20.3)	
Mild	65 (45.8)	19 (38.0)	84 (43.8)	
Moderate	29 (20.4)	10 (20.0)	39 (20.3)	
Marked	20 (14.1)	10 (20.0)	30 (15.6)	
ADC value [M(Q1~Q3),×10–^3^ mm^2^/s]	0.99 (0.87~1.09)	0.92 (0.79~1.03)	0.97 (0.84~1.06)	**0.027**
TIC type				0.532
Persistent	6 (4.2)	1 (2.0)	7 (3.6)	
Plateau	42 (29.6)	19 (38.0)	61 (31.8)	
Washout	94 (66.2)	30 (60.0)	124 (64.6)	
Rate of early enhancement				0.902
>120%	99 (69.7)	36 (72.0)	135 (70.3)	
≤120%	43 (30.3)	14 (28.0)	57 (29.7)	

LVI, lymphovascular invasion; IDC, invasive ductal carcinoma; pT, Pathological T stage of TNM; ER, estrogen receptor; PR, progesterone receptor; HER2, human epidermal growth factor receptor 2; SBR, Scarff-Bloom-Richardson; ALN, axillary lymph node; pN, Pathological N stage of TNM; BPE, background parenchyma enhancement; ADC, apparent diffusion coefficient; TIC, time-intensity curve.

Bold values indicate statistically significant differences.

### 3.2 Clinical-pathological features analysis

Compared to the LVI(-) group, the LVI(+) group had a higher proportion of patients under 45 years of age (52% *vs*. 33.1%, *P* = 0.028), larger tumors (2.7 cm *vs*. 2.0 cm, *P* = 0.007), and a statistically significant difference in pathological T stage (*P* = 0.030). In terms of IHC, the proportion of high Ki-67 expression was higher in the LVI(+) group (96.0% *vs*. 79.6%, *P* = 0.013), while no statistically significant differences were observed in ER status (*P* = 0.067), PR status (*P* = 0.142), or HER2 status (*P* = 0.649). When comparing molecular subtypes, the proportion of Luminal A subtype was lower in the LVI(+) group (4.0% *vs*. 19.7%, *P* < 0.019), while the proportion of triple-negative subtype was higher (12.0% *vs*. 5.6%, *P* < 0.019). Additionally, the LVI(+) group had a significantly higher rate of axillary lymph node positivity (80.0% *vs*. 38.7%, *P* < 0.001), and there was a statistically significant difference in pathological N stage (*P* < 0.001). No statistically significant differences were observed between the two groups in terms of neural invasion or SBR grading (*P* = 1.000; *P* = 0.696) (**Table 1**).

### 3.3 DCE-MRI features analysis

Compared to the LVI(-) group, the LVI(+) group had a lower median ADC value (0.92×10^−^³ mm²/s *vs*. 0.99×10^−^³ mm²/s, *P* = 0.027). No statistically significant differences were observed between the two groups in terms of internal enhancement patterns (*P* = 1.000; *P* = 0.172; *P* = 0.987; *P* = 0.507), distribution patterns (*P* = 0.070), BPE (*P* = 0.697), TIC types (*P* = 0.532), or early enhancement rate (*P* = 0.902) (**Table 1**).

### 3.4 Risk factors of LVI in patients with invasive breast cancer

**Figure 1:**
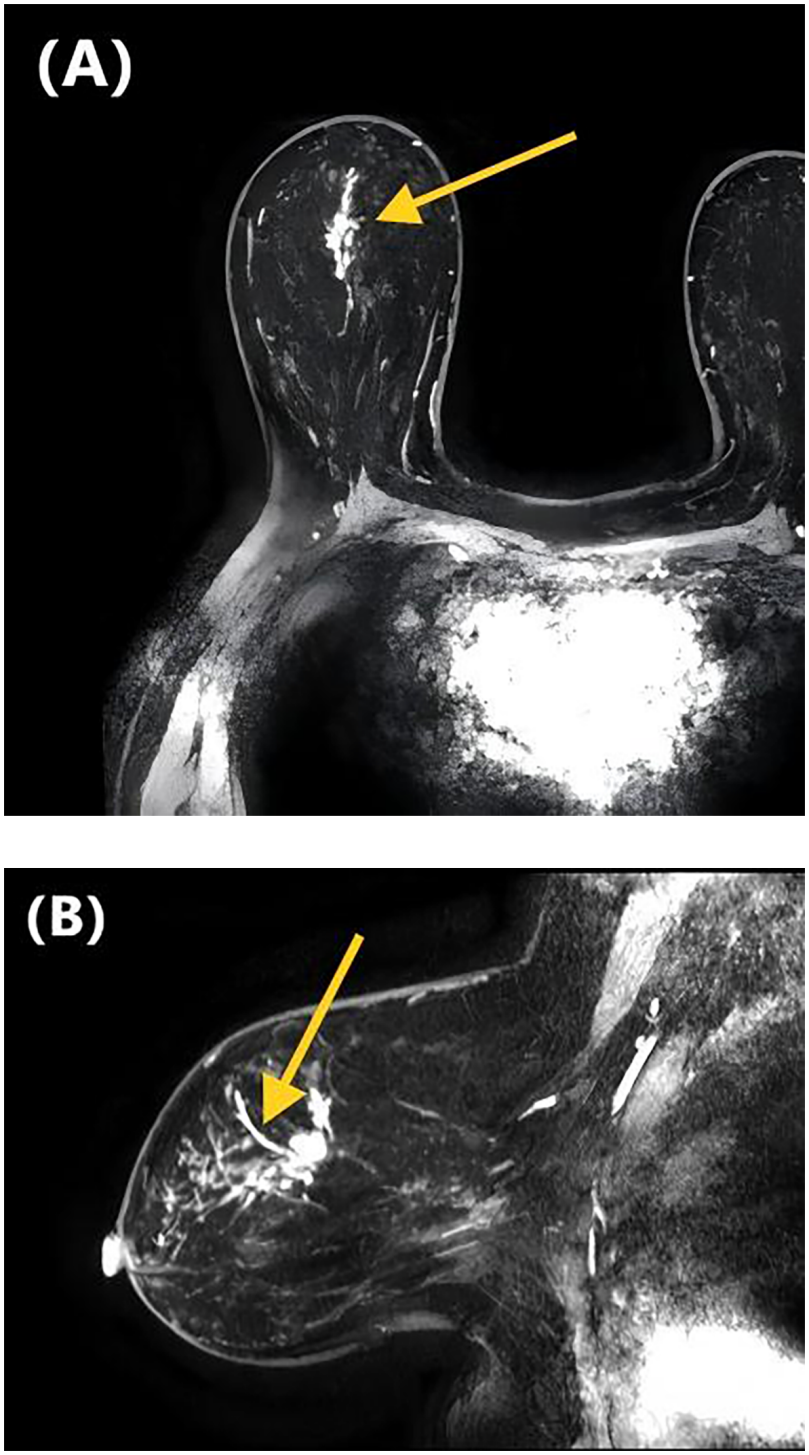
DCE-MRI images of a 47-year-old woman with invasive ductal carcinoma of the right breast. Axial contrast-enhanced image **(A)** and sagittal contrast-enhanced image **(B)** show non-mass-like enhancement with the linear distribution. Lymphovascular invasion was confirmed at the histopathological examination.

**Figure 2:**
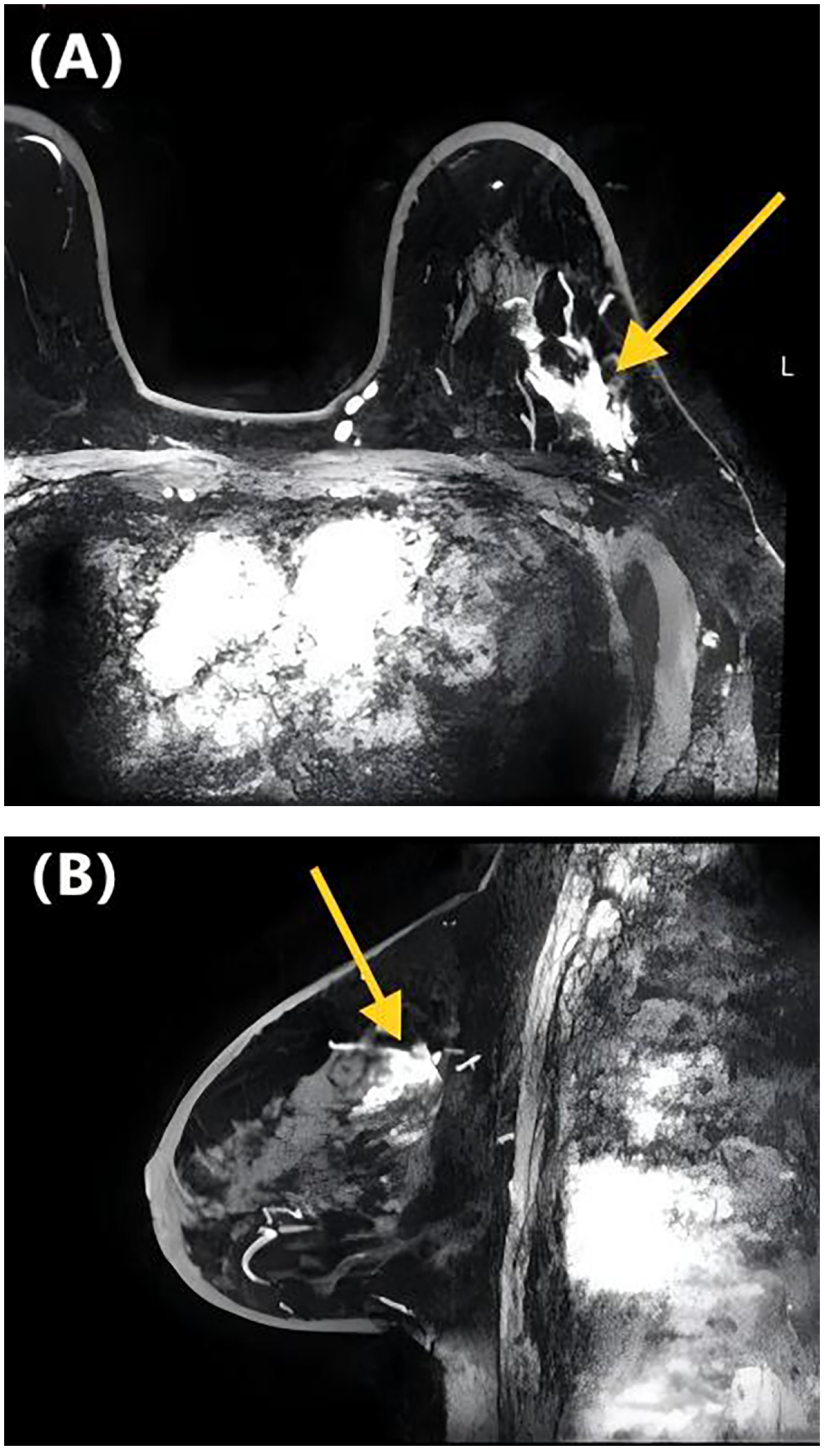
DCE-MRI images of a 65-year-old woman with invasive ductal carcinoma of the left breast. Axial contrast-enhanced image **(A)** and sagittal contrast-enhanced image **(B)** show non-mass-like enhancement with the focal distribution. Lymphovascular invasion was negative at the histopathological examination.

**Table 2** presents the GVIF analysis used to assess multicollinearity. The regression model demonstrated an absence of significant multicollinearity, as all adjusted GVIF values were below 2.24 except for PR status. Although the GVIF for PR status exceeded 2.24, it remained below the 3.16 tolerance threshold, indicating acceptable collinearity levels. After controlling for confounding factors through multivariate logistic regression analysis, four risk factors associated with LVI formation were identified (**Table 3**). Patients aged ≥45 years (*OR* = 0.406, 95% CI: 0.191-0.844, *P* = 0.017) and those with higher ADC values (*OR* = 0.133, 95% CI: 0.017-0.825, *P* = 0.041) had a lower risk of LVI formation. Among the NME distribution patterns, patients with linear distribution had a significantly higher risk of LVI positivity compared to those with focal distribution (*OR* = 13.540, 95% CI: 1.390-172.644, *P* = 0.030) (**Figures 1**, **2**), while no statistically significant differences were found for segmental, regional, multiple region, or diffuse enhancement patterns (all *P* > 0.05). Regarding molecular subtypes, compared to the Luminal A subtype, patients with Luminal B (*OR* = 5.081, 95% CI: 1.326-33.859, *P* = 0.039), HER2 overexpression (*OR* = 9.378, 95% CI: 1.922-71.460, *P* = 0.012), and triple-negative subtype (*OR* = 11.599, 95% CI: 2.043-96.710, *P* = 0.010) had a significantly higher risk of LVI formation.

**Table 2:** GVIF values for multicollinearity assessment.

Variables	GVIF	Df	GVIF^(1/(2*Df))^
Age	1.377	1	1.173
Homogeneous	1.161	1	1.078
Heterogeneous	2.161	1	1.470
Consecutive nodular	1.877	1	1.370
Agglomerated annularly	2.224	1	1.491
Distribution	2.859	5	1.111
BPE	1.723	3	1.095
ADC value	1.382	1	1.175
TIC type	1.493	2	1.105
Rate of early enhancement	1.385	1	1.177
Invasive carcinoma size	1.361	1	1.166
ER status	4.651	1	2.157
PR status	5.247	1	2.291
Ki-67 status	4.918	1	2.218
HER2 status	3.126	2	1.330
Molecular subtype	56.420	3	1.958
Nerve invasion	1.312	1	1.145
Tumour type	3.458	1	1.860
SBR grade	3.914	2	1.407
Tumor site	1.297	1	1.139

BPE, background parenchyma enhancement; ADC, apparent diffusion coefficient; TIC, time-intensity curve. ER, estrogen receptor; PR, progesterone receptor; HER2, human epidermal growth factor receptor 2; SBR, Scarff-Bloom-Richardson.

**Table 3:** Risk factors of LVI in patients with invasive breast cancer.

Variables	*OR*	95%CI	*P* value
Age (≥45 yrs)	0.406	0.191-0.844	**0.017**
Distribution			
Focal	Ref.	–	–
Linear	13.540	1.390-172.644	**0.030**
Segmental	1.832	0.430-12.724	0.463
Regional	3.327	0.642-25.678	0.183
Multiple regional	2.528	0.333-23.250	0.370
Diffuse	5.139	0.975-40.537	0.074
ADC value	0.133	0.017-0.825	**0.041**
Molecular subtype			
Luminal A	Ref.	–	–
Luminal B	5.081	1.326-33.859	**0.039**
HER2-overexpression	9.378	1.922-71.460	**0.012**
Triple-negative	11.599	2.043-96.710	**0.010**

LVI, lymphovascular invasion; ADC, apparent diffusion coefficient.

Bold values indicate statistically significant differences.

### 3.5 Diagnostic performance of each risk factor for predicting LVI

**Figure 3:**
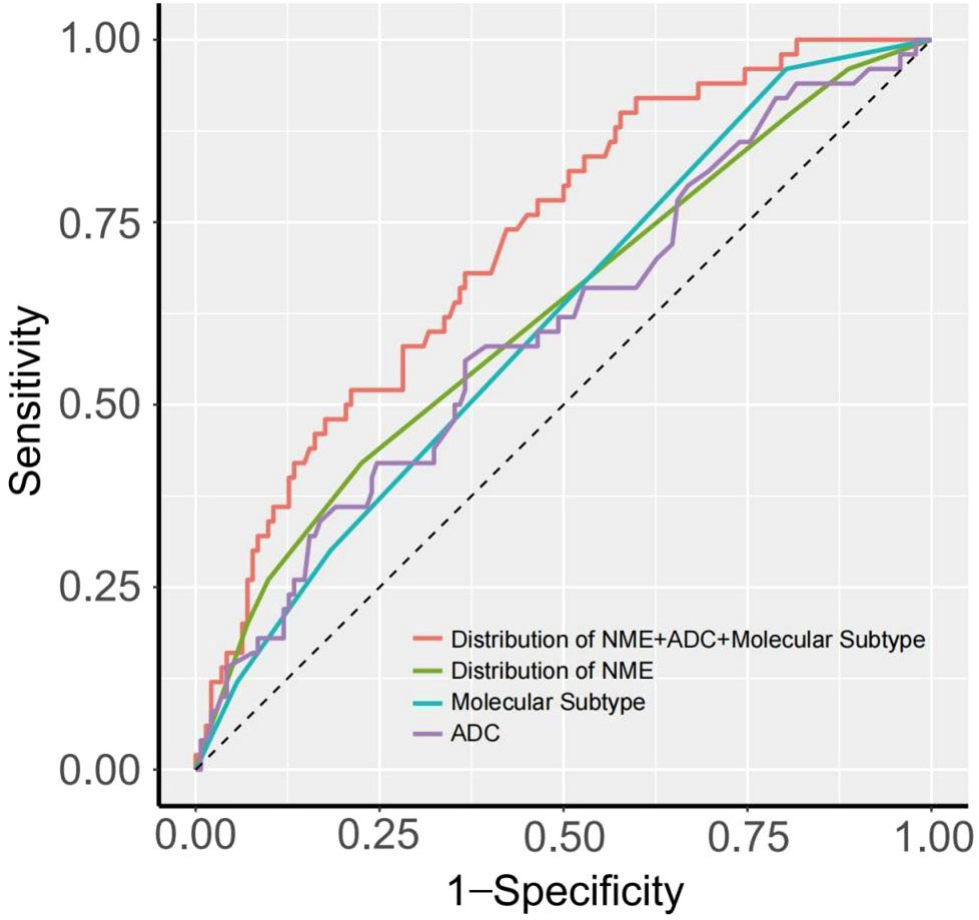
ROC curve of each risk factor predicting LVI.

We evaluated the diagnostic performance of each risk factor for predicting LVI using ROC curves, and the DeLong test was applied to compare differences in AUC values. **Figure 3** and **Table 4** present the diagnostic performance of Distribution of NME + ADC + Molecular subtype and other individual risk factors for predicting LVI.

**Table 4:** Comparison of AUC values of each risk factor for predicting LVI.

Risk factors	AUC	95% CI	*P* value
Distribution of NME + ADC + Molecular subtype	0.723	0.645-0.802	Ref.
Distribution of NME	0.625	0.540-0.709	**0.008**
Molecular subtype	0.614	0.540-0.687	**0.006**
ADC	0.606	0.513-0.698	**0.012**

LVI, lymphovascular invasion; AUC, Area under the curve; NME, non-mass enhancement; ADC, apparent diffusion coefficient.

Bold values indicate statistically significant differences.

**Figure 4:**
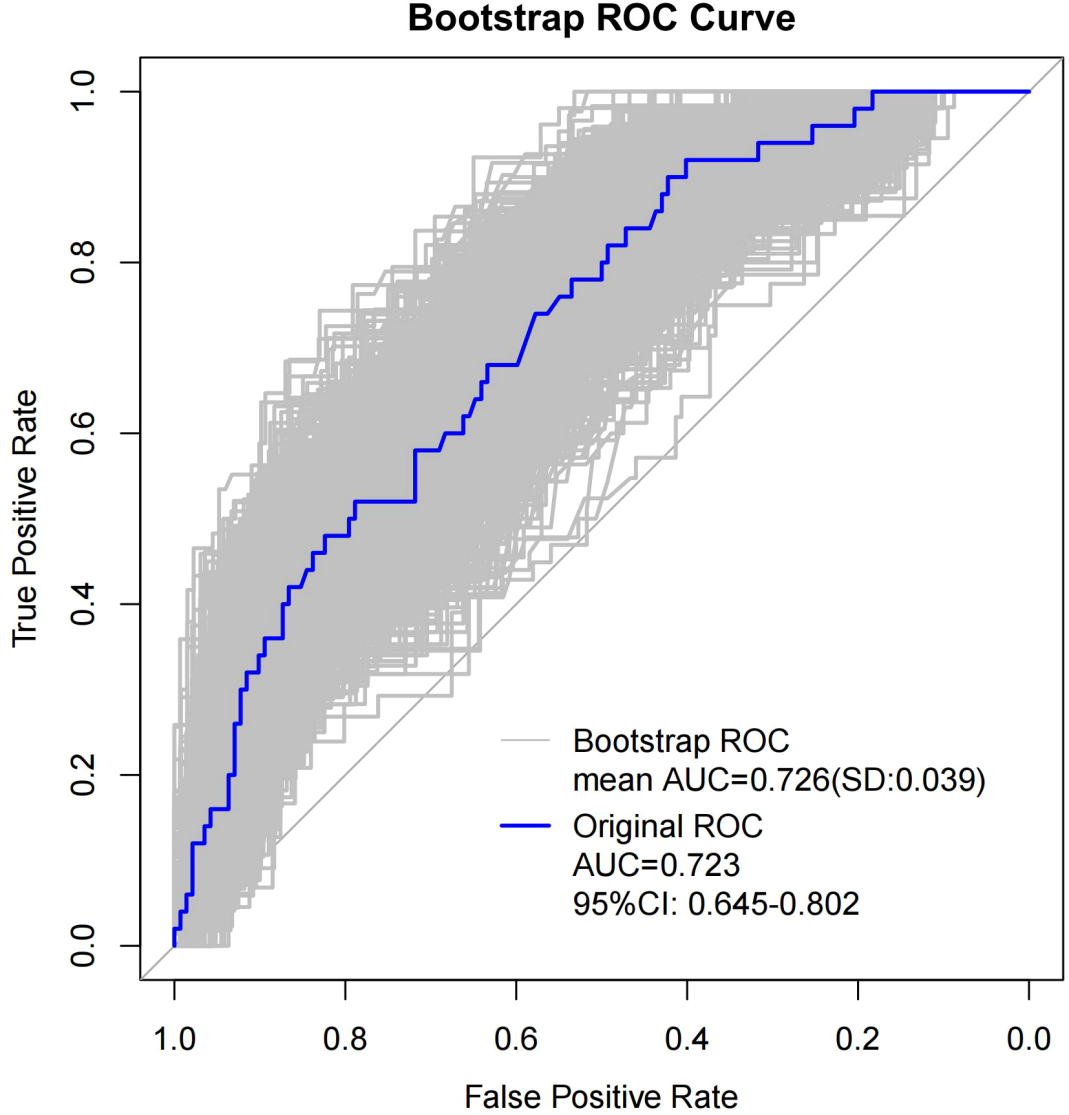
Resampling-corrected ROC for overfitting.

**Figure 5:**
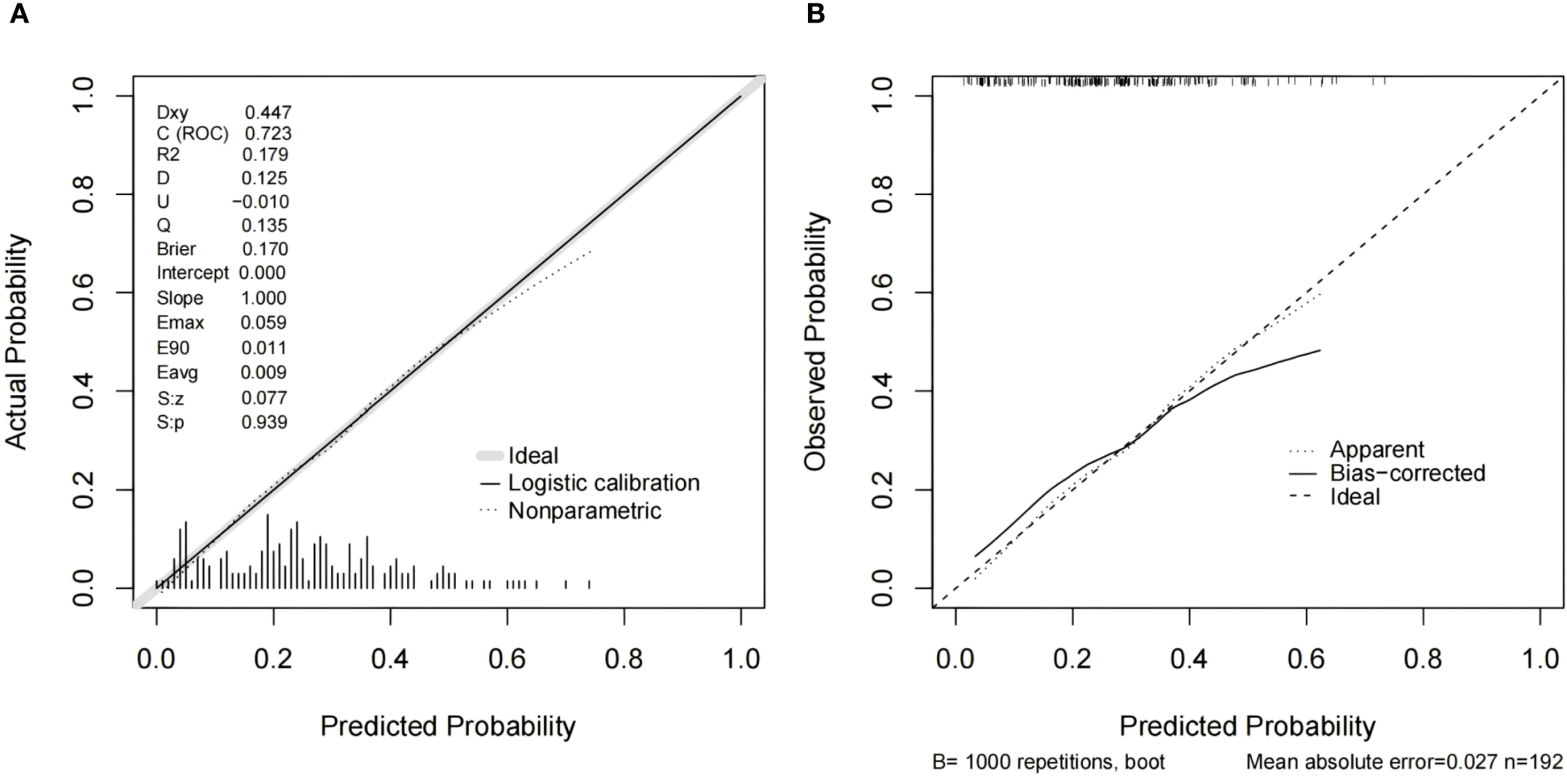
Calibration curves for the model integrating Distribution of NME + ADC + Molecular subtype **(A)** and resampling-corrected calibration curve for overfitting **(B)**.

**Figure 6:**
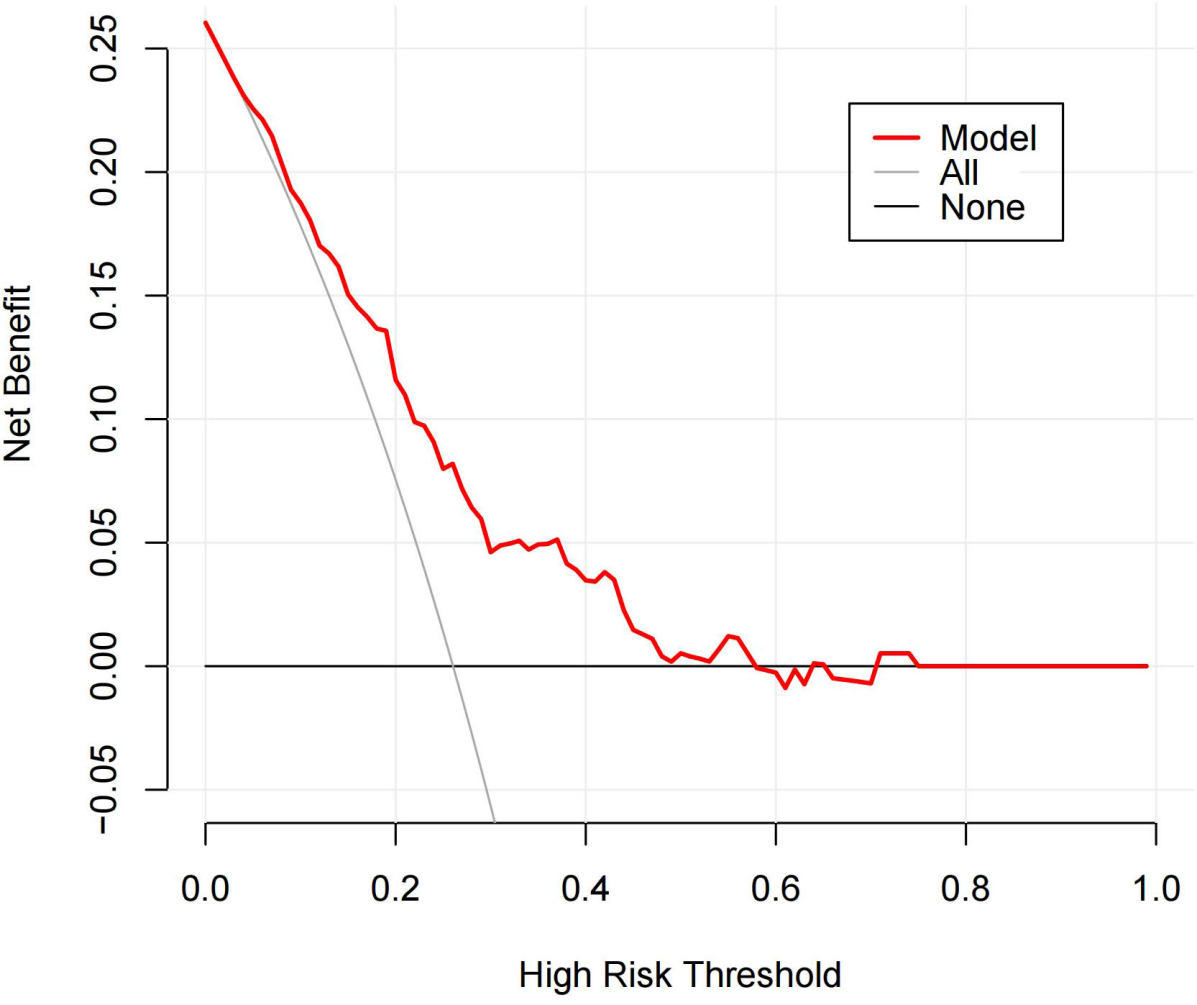
DCA of the Distribution of NME + ADC + Molecular subtype prediction model. The y-axis represents net benefit. The solid red line denotes the predictive model incorporating Distribution of NME + ADC + Molecular subtype. The solid gray line indicates the ‘treat-all’ strategy (assuming all patients belong to the LVI-positive cohort). The solid black line represents the ‘treat-none’ strategy (assuming no patients are included in the LVI-positive cohort). The x-axis displays threshold probability, defined as the probability at which the expected benefit of treatment equals the expected benefit of avoiding treatment. The DCA demonstrates that the predictive model yields net benefit across clinically relevant threshold probabilities.

As shown in **Table 4**, when using only the Distribution of NME to predict LVI, the AUC was 0.625. However, when ADC and Molecular subtype were added, the AUC significantly increased to 0.723 (*P* = 0.008, DeLong test). Similarly, the AUCs for Molecular subtype and ADC alone were 0.614 and 0.606, respectively. When compared to the AUC of Distribution of NME + ADC + Molecular subtype, the differences were statistically significant (*P* = 0.006, *P* = 0.012, DeLong test). To control Type I error across the 3 comparisons, Bonferroni correction was applied with an adjusted significance threshold of α = 0.0167. All post-comparison *P* values remained below this adjusted α, indicating statistically significant differences after correction. The bootstrap-derived AUC (1,000 iterations) showed high concordance with the apparent AUC upon internal validation (**Figure 4**). The calibration plot revealed good predictive accuracy between the actual probability and predicted probability (**Figure 5**). The DCA demonstrates that the predictive model yields net benefit across clinically relevant threshold probabilities (**Figure 6**). These indicate the potential utility of Distribution of NME + ADC + Molecular subtype in predicting LVI. Additionally, compared to the basic model (ADC + Molecular subtype), the inclusion of Distribution of NME improved the model’s reclassification ability, with a NRI of 0.389 (95% CI: 0.083-0.696) and an IDI of 0.047 (95% CI: 0.012-0.083), as shown in **Table 5**. These findings demonstrate that the inclusion of Distribution of NME significantly enhances the predictive ability of the model for LVI.

**Table 5:** Model performance after the addition of Distribution of NME to the basic model (ADC + Molecular subtype) for predicting LVI.

Models	NRI	95% CI	*P* value	IDI	95% CI	*P* value
ADC + Molecular subtype	–	–	–	–	–	–
ADC + Molecular subtype +Distribution of NME	0.389	0.083-0.696	0.013	0.047	0.012-0.083	0.008

NRI, net classification improvement; IDI, integrated discrimination improvement; CI, confidence interval.

## 4 Discussion

The lesions of NME-type breast cancer are loose, often interspersed with normal fibroglandular tissue, making clinical palpation challenging. The pathology of NME-type breast cancer is diverse, and it lacks typical imaging features. Mammography and ultrasound have low sensitivity in detecting NME-type lesions, whereas DCE-MRI, with its high soft tissue and spatial resolution, allows for multiparametric and multidirectional imaging. This provides a detailed view of the morphological and hemodynamic characteristics of NME-type breast cancer, offering higher sensitivity. Due to the scattered nature of NME lesions, clinicians often approach breast-conserving surgery for NME-type breast cancer with caution, as these lesions may increase the risk of positive surgical margins, potentially affecting patient prognosis (13). Moreover, the St. Gallen Consensus reported that breast cancer patients with LVI positivity have a higher rate of local recurrence after breast-conserving surgery (14). Similarly, a study by LIU J et al. (13) found that LVI positivity is an independent predictor of positive surgical margins and an increased risk of local recurrence in breast-conserving surgery. Therefore, we speculate whether there are associated factors that may contribute to the increased risk of LVI in patients with NME-type breast cancer.

At present, the vast majority of research on NME lesions focuses on predicting malignancy or exploring ways to reduce unnecessary biopsies. As a highly specific form of breast cancer on DCE-MRI, the prognostic significance of NME-type breast cancer requires further investigation. LVI is a known risk factor for both local recurrence and distant metastasis in breast cancer patients, and it is also associated with poorer disease-free survival (DFS) and overall survival (OS) (15). Many studies have also identified other factors that influence breast cancer prognosis, such as age, axillary lymph node involvement, tumor histological grade, hormone receptor (HR) status, HER2 expression, and Ki-67 expression (16, 17). Some studies have reported correlations between LVI status and these established prognostic factors in breast cancer patients (18), however, results across different studies are not entirely consistent (19–21). Currently, there is limited research investigating the factors influencing LVI in NME-type breast cancer. This may be due to the relatively low incidence of NME, accounting for approximately 21% of invasive breast cancer cases (22). Our study focuses on this population, specifically exploring the relationship between DCE-MRI imaging features, clinical-pathological features, and LVI in patients with NME-type breast cancer.

In addition to local invasion, one of the key characteristics of cancer is its ability to spread, leading to the metastasis of cancer cells. Metastatic disease is the primary cause of death in cancer patients (23). Before cancer cells metastasize to secondary sites, they often first enter and spread through the lymphatic system. Therefore, LVI and minor perineural and neural invasion, is one of the biological prerequisites for systemic spread and metastasis (15). A study by Liu G et al. (24) reviewed the medical records of 14,782 unilateral breast cancer patients who underwent surgery at the Cancer Hospital of the Chinese Academy of Medical Sciences (CHCAMS) between January 2009 and September 2017. The study found that younger age was associated with poorer overall survival (OS), though the association with disease-free survival (DFS) was not significant. However, other studies have suggested that younger age is related to worse DFS (25, 26). These inconsistencies may be due to differences in histopathological types and staging. In our study, a higher proportion of patients in the LVI(+) group were younger than 45 years (52.0% *vs*. 33.1%, *P* = 0.028). Logistic multivariate regression analysis revealed that patients aged ≥45 years had a lower risk of LVI formation (*OR* = 0.406, 95%CI: 0.191-0.844, *P* = 0.017), which is consistent with previous studies (27, 28). It has been well-established that LVI positivity is a precursor for regional lymph node metastasis and an independent prognostic factor for distant metastasis in invasive breast cancer (3–5). Moreover, a study by Lee SJ et al. (29) demonstrated that LVI is also associated with higher recurrence rates and lower survival after surgery. Taken together with previous research and the findings from our study, these results suggest that the presence of LVI in younger breast cancer patients may be one of the factors contributing to their relatively poorer prognosis.

In terms of clinical-pathological features, CHOI BB’s study (30) suggested that histological grade, Ki-67 expression, and HR status were not correlated with LVI. However, in our study, the proportion of high Ki-67 expression was significantly higher in the LVI(+) group compared to the LVI(-) group (96.0% *vs*. 79.6%, *P* = 0.013). This discrepancy may be due to the smaller sample size in CHOI BB’s study, which included only 132 patients. On the other hand, a study by ABE H et al. (31) yielded results similar to ours, finding that LVI was associated with high tumor cell proliferation and high Ki-67 expression. Our study also found that, compared to the Luminal A subtype, patients with Luminal B, HER2 overexpression and triple-negative breast cancer had a higher risk of LVI formation. Previous studies have reported that LVI is more common in HR-negative, HER2 positive, and high Ki-67 expression tumors (32–34). According to the major intrinsic biological subtype of breast cancer, LVI is more common in HER2-overexpression and Luminal B subtypes (32, 34), which is similar to the results of our study. Furthermore, Van den Eynden et al. (35) found that LVI was significantly associated with lymph node metastasis, a conclusion also supported by SHEN SD et al. (18). In our study, the LVI(+) group had a higher rate of axillary lymph node positivity (80.0% *vs*. 38.7%, *P* < 0.001). Axillary lymph node metastasis often indicates an increased risk of distant metastasis, which helps explain why LVI is a risk factor for distant metastasis and is associated with poorer DFS and OS outcomes (13). Therefore, for patients diagnosed with invasive breast cancer via core needle biopsy, assessing HR, HER2 status, and Ki-67 expression in the biopsy tissue can help clinicians preliminarily determine the molecular subtype. This enables clinicians to be vigilant about the potential presence of LVI and select the more appropriate treatment strategy.

Most current research on NME detected by MRI focuses on distinguishing between benign and malignant lesions. Malignant breast tumors typically exhibit low ADC values, which is attributed to the increased number of cells, larger nuclei, abundant macromolecular proteins, and reduced extracellular space in malignant tumors, all of which significantly reduce the water molecule diffusion capacity. Additionally, ADC values may be affected by factors such as hemorrhage within the lesion, highly dense fluids, or enlarged lymph nodes in the breast (36). Considering the intermingling of tumors and normal tissues in NME lesions, the ADC value of lesions was obtained by selecting multiple points for the measurement and taking the average value to avoid bias in the data as much as possible. Some studies have shown that ADC values are related to tumor invasiveness in breast cancer patients and are associated with prognosis (37–39), other researches have demonstrated a negative correlation between ADC values and LVI (21, 40, 41). Retrospective cohort studies by DURANDO M et al. (42) and IGARASHI T et al. (43) both found that ADC values were significantly lower in the LVI-positive group compared to the LVI-negative group, which is consistent with our findings.

A study by CHIKARMANE SA et al. (44) found that the most common distribution pattern of NME-type breast cancer on DCE-MRI was focal (49.7%; 102/205), followed by linear (25.6%; 53/205). However, other studies have indicated that segmental distribution was more common (45, 46). The discrepancy in findings may be attributed to differences in study populations and sample sizes. In our study, regardless of LVI status, the most common distribution pattern was segmental. The reason for this result may be related to the growth of the tumor along the duct. After multivariate adjustment, we found that patients with linear distribution of NME had a significantly higher risk of LVI positivity compared to those with focal distribution (*P* = 0.030). Although the total number of cases with linear distribution in our data was small (N = 7), this result suggests a possible correlation between lesion distribution patterns and LVI formation in NME patients. In the future, we plan to collect more samples to further validate the reliability of this conclusion. To further investigate whether distribution patterns play a role in predicting LVI, we applied NRI and IDI for evaluation. The results demonstrated that the inclusion of Distribution of NME significantly improved the predictive ability of the basic model (ADC + Molecular subtype) for LVI [NRI (0.389; 95% CI: 0.083-0.696) and IDI (0.047; 95% CI: 0.012-0.083)]. Compared to the evaluation of LVI by individual risk factors, the AUC increased significantly to 0.723. Therefore, we believe that Distribution of NME may have potential utility in predicting LVI. Currently, no other studies have explored the relationship between distribution patterns of NME-type breast cancer and LVI, making our study somewhat novel in this regard. This may further alert clinicians to pay closer attention to NME-type breast cancer in order to develop more appropriate treatment strategies for patients at risk of LVI.

This study has several limitations. First, as a retrospective study, future prospective studies with larger sample sizes are needed to further validate our findings. Second, since this is a single-center study, there is a certain degree of selection bias in the enrolled patient population. Third, due to the relatively short follow-up period, we did not explore the relationship between NME and the prognosis of patients with invasive breast cancer. Lastly, the pathological types included in our study were limited to invasive carcinomas, meaning our sample does not fully represent the histological diversity of the general population.

## 5 Conclusions

Our study found that age ≥ 45 years and higher ADC values were associated with a lower risk of LVI formation. Among the NME distribution patterns, patients with linear distribution had a higher risk of LVI positivity compared to those with focal distribution. Regarding molecular subtypes, patients with Luminal B, HER2-overexpression, and triple-negative subtypes had a higher risk of LVI formation compared to those with Luminal A. Distribution of NME + ADC + Molecular subtype demonstrated relatively good predictive ability for LVI, with an AUC of 0.723, and the inclusion of Distribution of NME significantly improved its predictive capability. This study provides a reference for evaluating LVI during the preoperative imaging stage, contributing to clinical decision-making and personalized treatment.

## Data Availability

The raw data supporting the conclusions of this article will be made available by the authors, without undue reservation.
